# PET Imaging of Cardiac Hypoxia: Hitting Hypoxia Where It Hurts

**DOI:** 10.1007/s12410-018-9447-3

**Published:** 2018-02-23

**Authors:** Victoria R. Pell, Friedrich Baark, Filipa Mota, James E. Clark, Richard Southworth

**Affiliations:** 1 School of Biomedical Engineering and Imaging Sciences, King’s College London, London, UK; 2 School of Cardiovascular Medicine and Sciences, BHF Centre, King’s College London, London, UK

**Keywords:** Positron emission tomography, Cardiac hypoxia, Cardiac ischemia, Copper bis(thiosemicarbazones), ^18^F-MISO, ^64^Cu-CTS

## Abstract

**Purpose of Review:**

In this review, we outline the potential for hypoxia imaging as a diagnostic and prognostic tool in cardiology. We describe the lead hypoxia PET radiotracers currently in development and propose a rationale for how they should most appropriately be screened and validated.

**Recent Findings:**

While the majority of hypoxia imaging agents has been developed for oncology, the requirements for hypoxia imaging in cardiology are different. Recent work suggests that the bis(thiosemicarbazone) family of compounds may be capable of detecting the subtle degrees of hypoxia associated with cardiovascular syndromes, and that they have the potential to be “tuned” to provide different tracers for different applications.

**Summary:**

New tracers currently in development show significant promise for imaging evolving cardiovascular disease. Fundamental to their exploitation is their careful, considered validation and characterization so that the information they provide delivers the greatest prognostic insight achievable.

## Introduction

Cardiovascular disease (CVD) is the leading cause of mortality worldwide, and its incidence is projected to rise significantly in the foreseeable future. There is a clear need for novel techniques which can aid in its diagnosis and treatment. Tissue hypoxia, as a component of ischemia, is a primary factor in conditions such as stroke and myocardial infarction and is thought to play a role in the structural and functional changes underlying chronic cardiovascular diseases such as coronary microvascular dysfunction (CMVD), cardiac hypertrophy, and heart failure [1, 2]. However, the precise relationship between intracellular oxygen concentration and the pathophysiology of cardiovascular disease in humans is poorly understood. This is due in part to the lack of techniques capable of non-invasively quantifying and characterizing tissue hypoxia in patients. Positron emission tomography (PET) is a relatively underexploited technology in cardiology which has the capacity to non-invasively report on intracellular biochemical events with exquisite sensitivity. In this review, we discuss the potential for utilizing PET imaging to quantify hypoxia in progressive cardiovascular disease, give an overview of the current status of hypoxia-specific PET probe development for cardiovascular applications, and suggest an approach for the screening and selection of future hypoxia imaging agents.

## Why Image Cardiac Hypoxia?

Cardiac metabolism is predominantly aerobic. As such, the maintenance of an adequate supply of oxygen to myocytes is fundamental to cardiac function and metabolism. Hypoxia, a condition in which intracellular oxygen is too low to satisfy metabolic demand, results in a downstream cascade of disturbances in cellular metabolism including the slowing of oxidative phosphorylation, a switch to anaerobic glycolysis, elevated lactate production, and changes in gene expression, mediated largely by hypoxia-inducible factor-1α (HIF-1α) [3, 4]. Diseases characterized by hypoxia benefit from early diagnosis and treatment in order to reduce mortality and morbidity. However, the imaging modalities most frequently used in cardiac imaging, echocardiography, and magnetic resonance imaging (MRI), largely report on physical changes in cardiac structure or contractile function. Subtle limitations in oxygen availability are likely to have biochemical implications for the myocardium long before the effects manifest as the structural or contractile abnormalities which are currently measured clinically, by which time the opportunity for optimal intervention may already have passed. Many cardiac pathologies exhibit low-grade hypoxia as part of their disease progression and could potentially benefit from such an early molecular imaging approach. Non-compensated cardiac hypertrophy is associated with impaired vascularization, resulting in cardiomyocytes that are increasingly vulnerable to ischaemia [1, 5]. Increases in cardiomyocyte size and progressive tissue fibrosis exacerbate the scenario by extending diffusion distances between blood vessels and mitochondria. This results in an imbalance between oxygen supply and demand (irrespective of concomitant coronary artery disease), leading to cardiomyocyte degeneration, and the inevitable transition to heart failure [1, 5]. Coronary microvascular dysfunction (CMVD) is common in patients with persistent microvascular angina, obesity, diabetes and hypertension, and underlies the no-reflow phenomenon following acute myocardial infarction [6, 7]. Despite its prevalence in a multitude of clinical scenarios, including the progression of cardiomyopathy, it remains difficult to definitively diagnose because current non-invasive techniques lack the necessary resolution to visualize the coronary microcirculation or quantify perfusion at the microvascular level [8, 9]. Diagnosis of CMVD in patients is therefore typically made through the exclusion of other cardiomyopathies [2]. Heart failure with preserved ejection fraction (HFpEF) is a similarly poorly understood phenomenon. Representing approximately 50% of all heart failure cases, it is a significant cause of mortality and morbidity and is expected to become more prevalent as life expectancies continue to rise [10]. The mechanistic causes behind HFpEF are highly multi-factorial, making it difficult to classify, diagnose, and treat [11, 12], but microvascular dysfunction has been suggested to play a role [13, 14]. For these syndromes characterized by diffuse low-grade ischemia, often in the absence of a measurable gross perfusion deficit, a definitive, quantifiable, and easily interpreted readout of regional oxygen insufficiency could allow an early and unequivocal diagnosis, as well as rapid feedback on the effectiveness of new pharmacological or surgical interventions [15]. Imaging the tissue hypoxia which arises as a direct result of microvascular occlusion could provide a positive diagnostic test for CMVD and HFpEF without the requirement for high-resolution imaging of the microvessels themselves.

## Imaging Strategies for Myocardial Oxygenation

There are several clinically available approaches capable of reporting upon myocardial oxygenation, either non-invasively (blood oxygen level-dependent (BOLD) MRI, single-photon emission computed tomography (SPECT), and PET) or invasively (oxygen probes and Doppler flow wires). BOLD MRI distinguishes paramagnetic deoxyhemoglobin from oxyhemoglobin, revealing changes in vascular oxygenation [16, 17]. However, because BOLD only measures hemoglobin status in the bloodstream, the biochemical consequence on underlying tissues can only be inferred and not directly determined. Invasive procedures, via the use of oxygen-sensitive electrodes, are also limited both in terms of capacity and practicality. While they have been used for quantifying hypoxia within tumors, they are practically very difficult (and potentially dangerous) to apply in the heart. They provide only a measure of vascular or interstitial oxygen tension in a relatively small sample of tissue, with no spatial information, and their requirement for an accessible site further restricts their utility [18, 19].

The nuclear imaging techniques PET and SPECT provide real-time quantitative spatial information on the biodistribution of the injected radiolabeled tracer at sub-pharmacological (≥pM) concentrations. They allow the non-invasive imaging of biological systems with great sensitivity, without disrupting them [20]. Most existing cardiac PET techniques detect either regional perfusion heterogeneity or metabolic changes [21]. The metabolic tracers ^18^F-fluorodeoxyglucose (^18^FDG) and ^11^C-1-acetate, for example, report on myocardial glycolysis and the citric acid cycle respectively, but their specificity is limited in that their uptake is governed by numerous factors independent of hypoxia or ischemia, including blood flow, substrate availability, hormonal status, insulin sensitivity, inflammation, and cardiac workload [22, 23]. Similarly, most other nuclear cardiology approaches do not measure cellular hypoxia directly. These techniques, including ^15^O-water and ^13^N-ammonia PET perfusion tracers, infer hypoxia from abnormalities in perfusion and have evolved such that the evaluation of absolute coronary flow is now highly quantitative, offering real advantages in improving the diagnosis of CMVD patients [24•]. However, hypoxia is classically defined as a mismatch between oxygen supply and demand that has a pathophysiological consequence [25]; such measurements of flow alone cannot therefore fully account for the other factors that may influence oxygen delivery or diffusion such as blood oxygen content or alterations in cardiac structure [1, 26, 27]. Furthermore, while assessing cardiac perfusion is undoubtedly predictive of myocardial compromise, without reference to metabolic demand, it is not as informative as it could be. There consequently remains a clear need for a hypoxia imaging method that will provide the possibility of identifying cardiovascular disease earlier, visualizing ischemic syndromes which are currently undetectable, and assessing response to therapy.

While this review will be limited to recent developments in hypoxia PET probe development, numerous complexes have also been evaluated for imaging by SPECT, and readers are directed to previous reviews covering this in detail [15, 28, 29].

## PET Imaging of Myocardial Hypoxia

There are a number of characteristics that an ideal PET hypoxia probe should satisfy. Firstly, as the function of these imaging agents is to selectively identify hypoxic cells or regions, they must demonstrate high retention in hypoxic tissue but must also clear rapidly from both blood and non-target tissues to provide a high target-background image contrast, or signal-to-noise ratio (ideally above 3:1), independent of perfusion [30]. Hepatic clearance should also be low in order to minimize interference when quantifying uptake in the heart. This is frequently a trade-off in radiotracer selection; increased lipophilicity promotes cell penetration through diffusion but also results in higher non-selective tissue retention in cell membranes and higher liver uptake. Similarly, the redox potential of the radiotracer determines the hypoxia threshold at which it is reduced and trapped within the cell (or deposits its radiometal payload), and ultimately determines what imaging applications it may be suited for [31, 32]. Hypoxia tracer development therefore requires a balance to be struck between lipophilicity and redox potential to obtain optimal hypoxia selectivity, sensitivity, and appropriate pharmacokinetics.

Over the decades a number of PET tracers have been developed for the identification of hypoxia, mostly with cancer imaging applications in mind. Because of low oxygen requirements and chaotic vasculature common in tumors, the degrees of hypoxia common in tumors are far more extreme than would ever be present in hypoxic but potentially salvageable myocardium [25, 33]. It is therefore unlikely that hypoxia imaging agents optimized for cancer imaging would be equally well suited to imaging cardiovascular disease. Significant refinement of existing hypoxia tracer design is therefore required for cardiovascular application, and the development of more suitable radiotracers is ongoing.

## A Suggested Approach for Hypoxia Imaging Agent Screening—“Hitting It Where It Hurts”

For cardiovascular applications, it is essential to target tracer selectivity to the degrees of hypoxia prevalent in chronically ischemic myocardium clinically, rather than quantifying maximal uptake during anoxia or extreme hypoxia, which has been the historical approach in cell cultures or isolated perfused hearts. While determining gross tracer uptake during anoxia is a useful first screen for identifying hypoxia-sensitive tracer candidates, it is not safe to assume that the hypoxic response for all tracers is linear or that tracers which are retained during anoxia are more sensitive than their counterparts across all degrees of hypoxia. Comparative hypoxia sensitivity titrations are clearly necessary, but this then raises the question of “what level of hypoxia is pathologically relevant (i.e., what do we want our imaging agent to identify for us), and how do we incorporate that information in a screen?” Perfusion of hearts with hypoxic buffers with no metabolic context is of limited use, and readouts of functional or contractile response are largely only relevant to the experimental model, rather than the clinical situation. Similarly, since intracellular oxygen concentration in cardiac pathologies is unknown (and therefore impossible to replicate experimentally), correlating tracer retention with titrated buffer oxygen concentrations provides limited further insight, particularly since blood and crystalloid buffer oxygen dissociation curves differ widely [34]. We would therefore suggest that when modeling hypoxia for screening imaging agents against chronic cardiovascular disease, degrees of hypoxic or ischemic compromise should be induced which invoke physiological or biochemical indices of injury representative of those known to exist clinically, namely decreased but not depleted PCr/ATP ratios, elevated lactate washout, and stabilization of HIF1-α [35–37]. While these all are established clinical biomarkers of ischemically compromised myocardium, they are invasive measures, or in the case of ^31^P MR spectroscopy require significant technical expertise and infrastructure which is not widespread, making them unlikely to become routine clinical tests in the foreseeable future. Identifying a hypoxia-selective PET imaging agent which is trapped at the threshold at which these biomarkers appear would provide rapid and easily interpretable insight into the biological status of the myocardium. Further, the use of hypoxia imaging agents validated against these biomarkers would give rise to a more informative range of imaging agents targeted at specific disease processes, beyond simplistic “hypoxic/normoxic” readouts. This approach could be expanded for use in other applications such as oncology, where hypoxia tracers are screened in cell or tissue preparations maintained at hypoxia thresholds associated with propensity to metastasize [38] or resistance to radiotherapy [39]. By validating in vitro or in vivo experiments with therapeutically relevant parallel biomarkers, they could be used to provide diagnostically or prognostically useful information beyond arbitrarily labeling tumors as “hypoxic” or “normoxic.”

## PET Hypoxia Tracers

In the search for hypoxia-specific tracers for cancer and cardiovascular applications, two classes of compounds have emerged; the nitroimidazoles and the copper bis(thiosemicarbazone) complexes (Cu-BTSCs).

### Nitroimidazoles

2-Nitroimidazoles were first developed as selective radio-sensitizing agents for the treatment of tumors with a hypoxic core [30]. As they are capable of selectively accumulating in hypoxic tissue, their basic design has served as a platform for radiotracers for both PET and SPECT imaging [40].

**Fig. 1:**
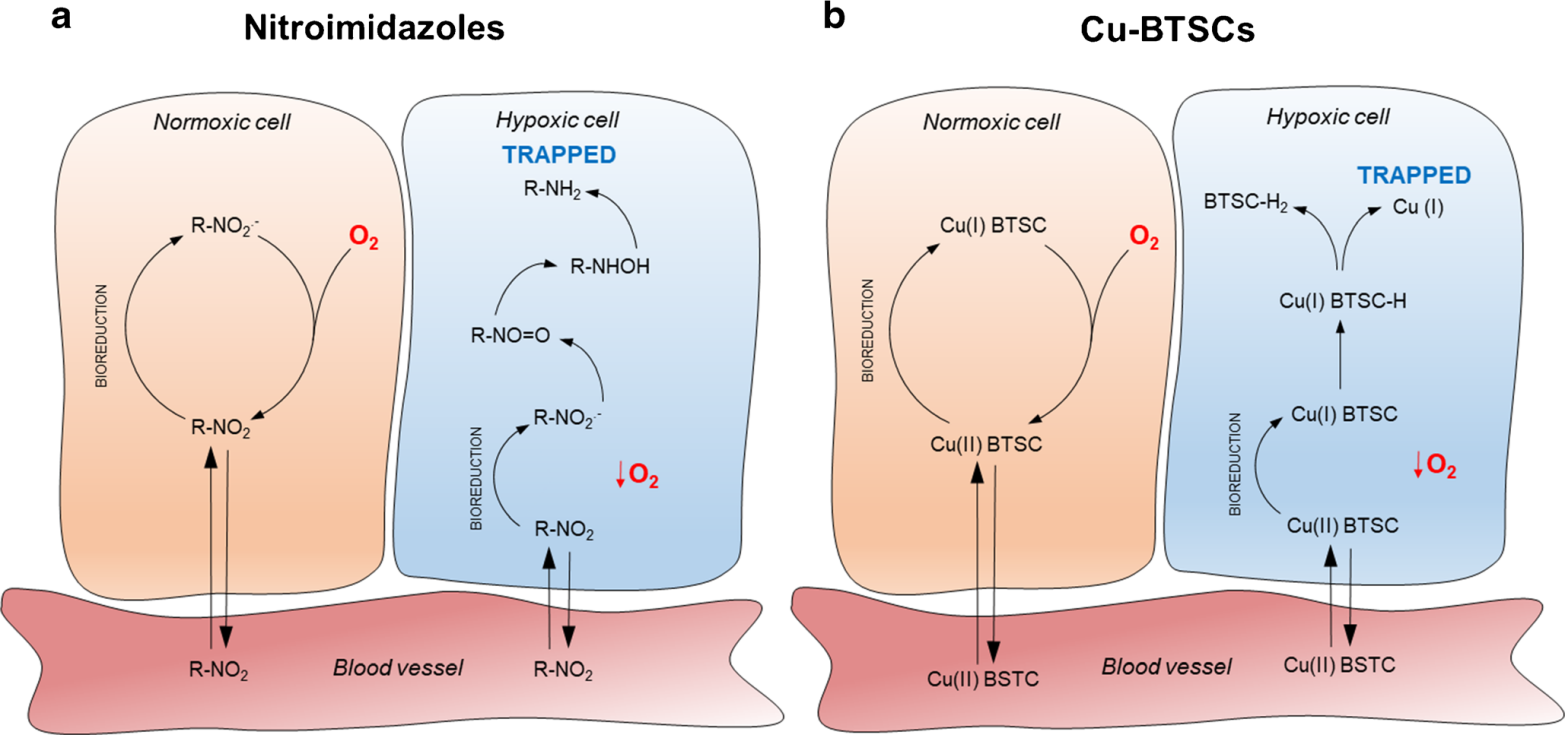
Simplified schematic of the proposed trapping mechanisms for nitroimidazole derivatives and Cu-BTSC PET tracers. **a** Upon entering the cell by diffusion nitroimidazoles become bio-reduced intracellularly to form nitro anion radicals (RNO_2_^**·−**^). In a normoxic cell, they are rapidly re-oxidized back to their uncharged form and are able to diffuse back out of the cell into the circulation. However, in a hypoxic environment, this oxidation is not possible and the radical anion is reduced further producing nitroso compounds (R–NO=O), hydroxylamines, and amines which can covalently bind intracellular macromolecules and become trapped. *R* denotes the radiolabeled species. **b** Cu(II)-BTSCs similarly diffuse into cells where they can be reduced to a charged Cu(I) complex which is unable to leave the cell. In the presence of oxygen, this Cu(I) complex is rapidly re-oxidized back to Cu(II) which is again able to diffuse out of the cell. If oxygen is insufficient, however, the Cu(I) complex can become further reduced and dissociate. The Cu(I) then becomes sequestered by copper chelating proteins and trapped inside the cell (Adapted with permission from Handley et al. [25])

Entering the cell by passive diffusion, nitroimidazole derivatives become bio-reduced intracellularly to form nitro anion radicals (RNO_2_^**·−**^), independent of oxygen tension. In the presence of oxygen, these anion radicals are rapidly re-oxidized back to their uncharged form and are able to diffuse back out of the cell into the circulation, with their rate of oxidation being dependent on intracellular oxygen concentration. In a hypoxic environment, the potential for re-oxidation is decreased, and the radical anions become reduced further in a step-wise manner to nitroso compounds, hydroxylamines, and amines [28]. These final products have a lower cell permeability and can covalently bind to macromolecules, resulting in them being selectively retained within hypoxic cells (Fig. 1a).

Within the nitroimidazole family of radiopharmaceuticals, ^18^F-fluoromisonidazole (^18^FMISO) was the first and most widely studied hypoxia-imaging probe for PET diagnosis, with its first clinical evaluation for cancer applications being reported in 1996 [41]. Its use in the heart has, however, been somewhat limited. ^18^FMISO has been demonstrated to selectively trap in hypoxic cardiomyocytes [42] and the canine ischemic myocardium following ligation of the left anterior descending coronary artery (LAD) [43, 44] (Table 1). These are, however, extreme models of hypoxia and are of questionable relevance to the clinic. More recently, the use of ^18^FMISO has shown promise in the detection of atherosclerotic plaques in rabbits and humans [45, 47] and even in the identification of human cardiac sarcoidosis [46]. Overall, however, the relative success that ^18^FMISO has had in cancer has failed to translate to the cardiac field. This may in part be explained by its limitations; ^18^FMISO is characterized by low first-pass uptake, slow blood clearance and high liver uptake, resulting in a low target-to-background image contrast of only 2:1. Patients must therefore be injected several hours prior to imaging to allow for blood clearance, and with an ^18^F half-life of only 110 min, a high initial dose is necessary [59]. Next-generation nitroimidazole PET tracers are being developed, including ^18^F-fluoroazomycin arabinoside (^18^F-FAZA), ^18^F-2-(2-nitro-1H-imidazol-1-yl)-*N*-(2,2,3,3,3-pentafluoropropyl)-acetamide (^18^F-EF5) and ^18^F-2-(4-((2-nitro-1H-imidazol-1-yl)methyl)-1H-1,2,3-triazol-1-yl)propan-1-ol (^18^F-HX4), with the aim of improving blood clearance and image contrast [49, 60]. Whether these prove to have more success than ^18^FMISO in cardiac disease remains to be seen, but an important question yet to be addressed is whether any agent within this class of compounds are sufficiently sensitive to the subtle degrees of hypoxia associated with compromised myocardium, rather than the extreme tumor hypoxia that they were originally designed to target.

**Table 1:**
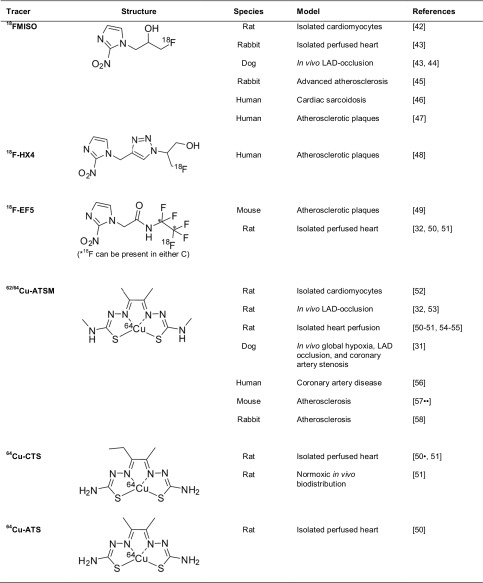
A selection of hypoxia-sensitive PET tracers and their structure and cardiovascular models in which they have been used

### Bis(thiosemicarbazones)

Cu-BTSCs show significant promise as PET imaging agents. With a half-life of 12.7 h, ^64^Cu can be conveniently shipped between sites and does not require an on-site cyclotron [27–29], while ^62^Cu, with a half-life of 9.7 min is produced by a portable generator [61], allowing flexibility with regard to labeling and imaging strategies. The lipophilic Cu(II)-BTSC complexes freely diffuse into the cell, where they become reduced to Cu(I)-BTSC intermediates. If sufficient oxygen is available, they are rapidly re-oxidized by molecular oxygen and are able to leave the cell intact. If intracellular oxygen levels are insufficient to re-oxidize the complex, it dissociates, releasing the radiocopper payload into the cell, where it is sequestered, giving rise to a hypoxia-dependent PET signal [62–64] (Fig. 1b). Cu-diacetyl-bis(N4-methylthiosemicarbazone) (Cu-ATSM) was the first BTSC demonstrated to exhibit hypoxia selectivity and is the most widely evaluated complex for both tumor [65] and cardiac hypoxia imaging [25]. It is highly cell permeable and clears rapidly from both normoxic tissues and blood [32, 53] generating high contrast images within minutes (compared with several hours when using ^18^FMISO). ^64^Cu-ATSM has been shown to deposit ^64^Cu in both hypoxic and ischemic isolated perfused hearts [50•, 51, 54], as well as in the in vivo regionally occluded canine myocardium [31]. In the one small clinical trial that we are aware of, Takahashi et al. compared the cardiac retention of ^62^Cu-ATSM and ^18^FDG in seven patients with coronary artery disease [56]. Of these, six had prior infarcts but were clinically stable, while the seventh had unstable angina. ^18^FDG PET imaging delineated regions of increased cardiac glucose metabolism in five patients, but ^62^Cu-ATSM retention was only observed in the patient with unstable angina [56]. Taken together, these studies suggest that while Cu-ATSM can detect extreme degrees of hypoxia in tumors and experimental models of extreme cardiac ischemia [62], it may not be sensitive enough to detect the subtle hypoxia thought to characterize chronic cardiac ischemic syndromes. By modifying their ligand backbone, however, the reduction potential of the Cu-BTSC complexes can be altered in order to deposit radiocopper in cells at different degrees of hypoxia, while properties important to optimizing imaging quality, such as lipophilicity or serum protein affinity, can be controlled by differently alkylating their terminal amino groups [31, 66–68]. There is, therefore, a potential to create a large catalog of structurally related analogs of Cu-ATSM that may be better suited to imaging cardiac hypoxia. We have embarked upon a program of designing, screening, validating, and characterizing such analogs and have thus far identified two complexes in this family, ^64^Cu-CTS and ^64^Cu-ATS which may be an improvement on Cu-ATSM for cardiovascular applications [50•, 51, 52].

### ^64^Cu-CTS—a Promising New Candidate

**Fig. 2:**
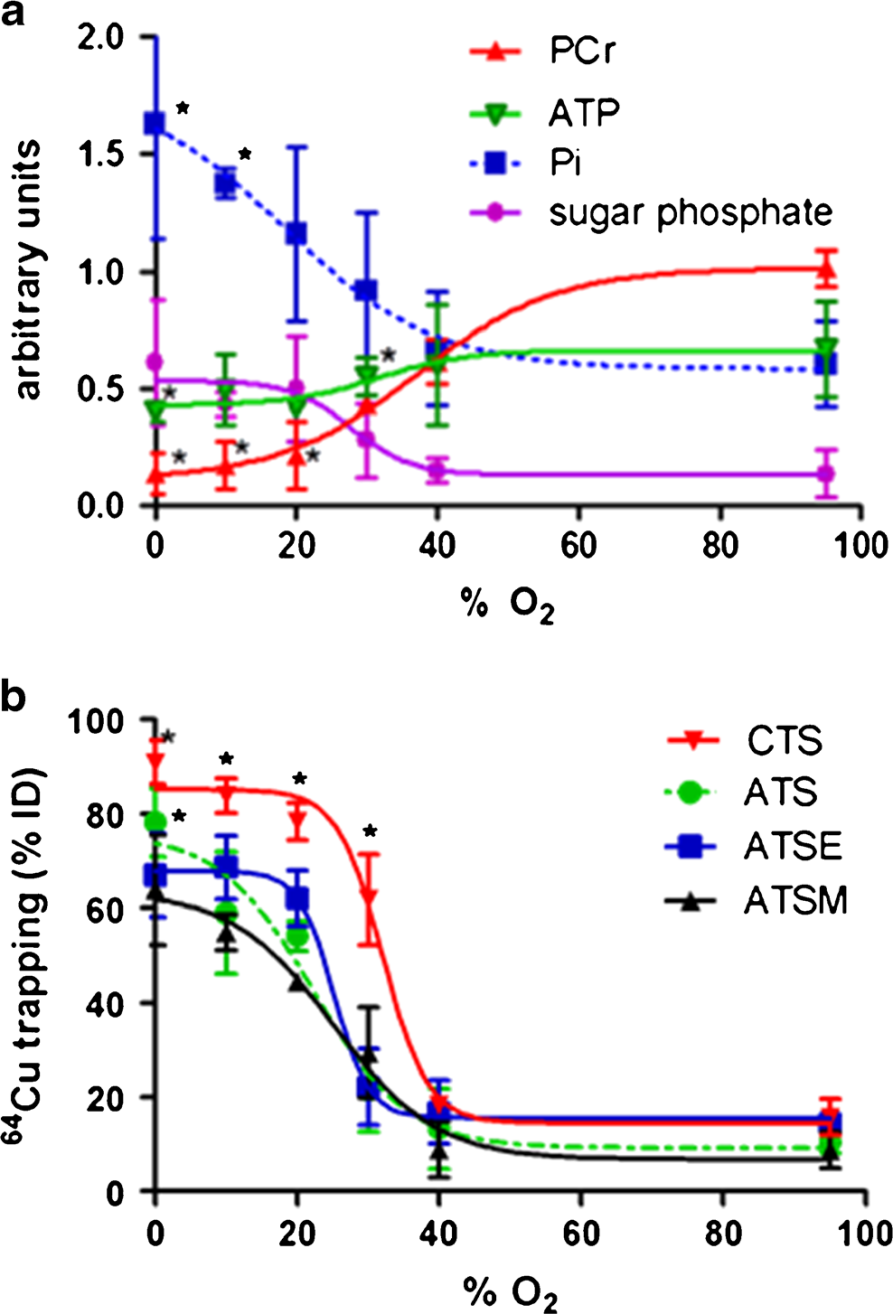
**a** The relationship between hypoxic buffer perfusion and cardiac energetics. Rat hearts were perfused for a stabilization period of 20 min, and then a range of hypoxic buffers for 45 min. ^31^P NMR spectra were acquired throughout. Data represent changes in phosphocreatine (PCr), ATP, inorganic phosphate (Pi), and sugar phosphates 25 min after the induction of hypoxia. *p < 0.05, significantly different from pre-hypoxic control. **b** The relationship between hypoxic buffer perfusion and ^64^Cu radiotracer uptake in hearts perfused in the same manner as described in (**a**). After 25 min of hypoxia, the heart was injected with a bolus of a radiotracer (1 MBq in 100 ml KHB), and its pharmacokinetics followed through the heart by NaI radiodetection. Data represent ^64^Cu retention from each tracer within the heart at each stage for ^64^CuCTS, ^64^CuATS, ^64^CuATSE, and ^64^CuATSM as a percentage of injected dose 10 min after injection. Data represent mean (*n* = 6 ± SD); *p < 0.05, significantly different from pre-hypoxic control values. This research was originally published in Medina et al. [50•]

Our recent screening of our BTSC library has revealed two complexes, ^64^Cu-ATS and ^64^Cu-CTS, which deliver significantly greater hypoxic contrast than either ^64^Cu-ATSM or ^18^F-MISO in isolated hearts perfused with hypoxic buffer [50•]. In line with our “hitting it where it hurts” approach, we then performed a buffer oxygenation titration study to identify the degree of perfusion buffer hypoxia which invoked hypocontractility, compromised PCr levels but stable ATP levels (by ^31^P NMR spectroscopy) (Fig. 2a), elevated but not maximal lactate washout (indicating onset of anaerobic metabolism but some oxidative reserve), and no creatine kinase leakage—a phenotype typical of chronically ischemic myocardium clinically. These criteria were met when hearts were perfused with buffer saturated with a 30% O_2_ gas mix. At this key threshold, ^64^Cu-CTS deposited significantly more radiocopper into the heart than any other tracer tested (including ^64^Cu-ATSM (Fig. 2b) [50•]), highlighting it as potentially suitable for clinical exploitation.

## PET Imaging of Hypoxia in Coronary Atherosclerosis

An area of cardiovascular molecular imaging in which the use of PET is gaining prominence is in the imaging of coronary atherosclerosis, and readers are directed to several recent reviews discussing this in more detail than will be covered here [69–71]. Atherosclerosis is a chronic inflammatory disease of the coronary vasculature characterized by the formation of inflammatory cell and lipid-rich lesions or plaques [72, 73]. These plaques develop over years, slowly encroaching upon the lumen and narrowing the artery, producing symptoms with exercise. Both early and advanced plaques can become unstable and rupture, resulting in arterial blockages which are the primary cause of myocardial infarction and stroke.

An advanced molecular imaging approach that allows evaluation of the processes linked to plaque instability and the identification of vulnerable plaques would be beneficial, particularly in high-risk patients. Of the approaches currently under investigation, the targeting of vascular inflammation and calcification has dominated, with ^18^FDG uptake correlating strongly with plaque inflammation in both preclinical models and patients [74, 75]. However, ^18^FDG has many limitations in terms of sensitivity and specificity [23, 76]. Its uptake is governed by numerous factors including cell type, local hypoxia, and diet, which may interfere with image analysis [47, 77, 78]. Furthermore, the significant background uptake of ^18^FDG in a metabolically active heart renders the visualization of coronary arteries extremely challenging [79]. As a result, there is considerable effort underway to develop more specific PET tracers for targeting unstable coronary lesions.

**Fig. 3:**
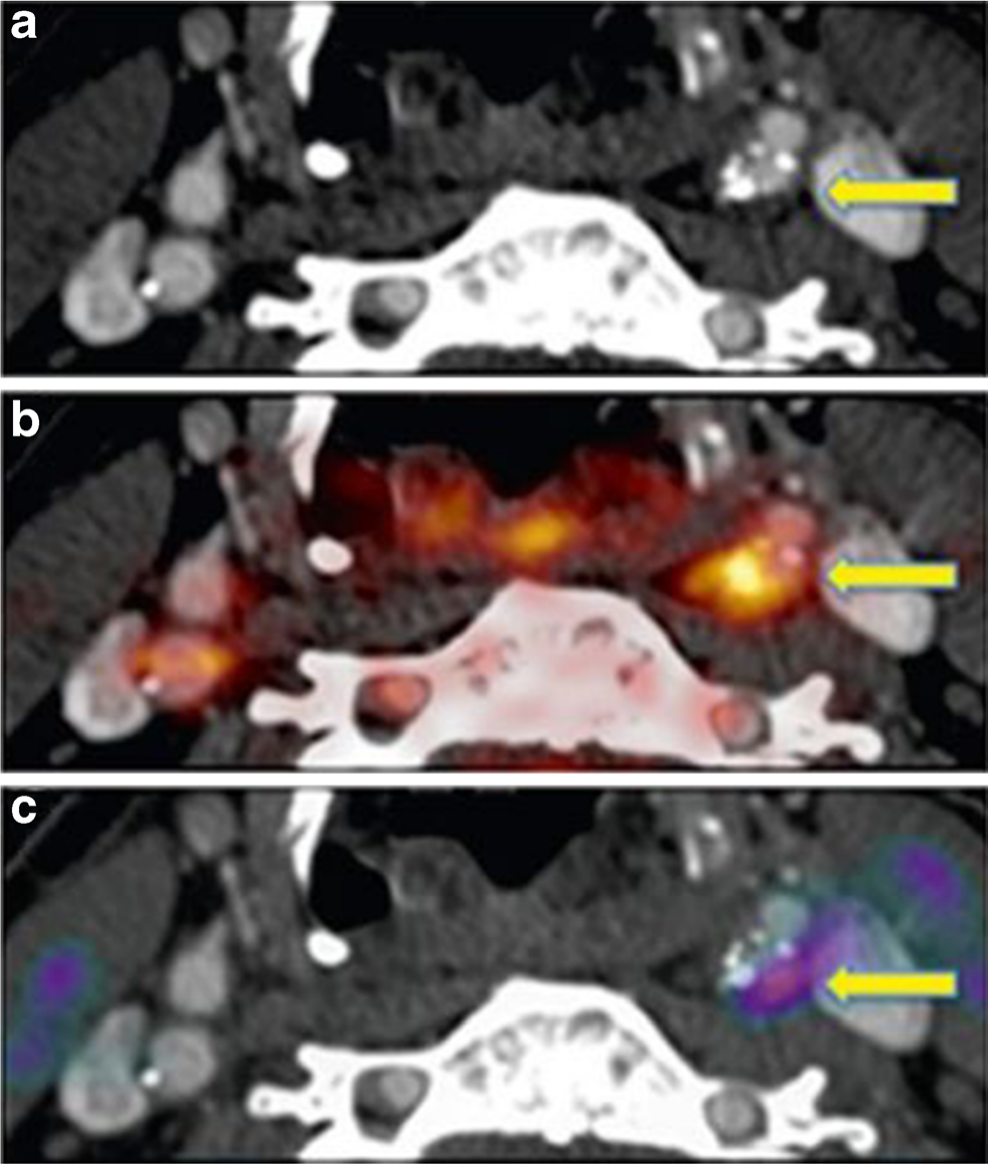
Positron emission tomography/computed tomography (PET/CT) imaging of a culprit carotid stenosis after stroke. **a** CT angiography. Stenosis in the left internal carotid artery (arrow). **b** Fused ^18^F-fluorodeoxyglucose (FDG) PET/CT. Intense uptake in the culprit lesion (arrow). **c** Fused ^18^F-fluoromisonidazole (FMISO) PET/CT. Corresponding uptake is indicative of intraplaque hypoxia (arrow). Reproduced from Joshi et al. [47]

Hypoxia is a characteristic feature of atherosclerotic plaque growth and has been linked to lesion progression through inflammatory cell activity and intraplaque angiogenesis [80–82]. It has also been suggested that hypoxia may contribute to the ^18^FDG signal observed from evolving plaques by stimulating macrophage and foam cell glucose uptake [47, 83]. A variety of PET hypoxia radiotracers, including those from both the nitroimidazole and BTSC families, have therefore been investigated as potential atheroma targeting imaging agents in experimental models. ^18^FMISO has been used to successfully visualize hypoxia in a rabbit model of advanced atherosclerosis [45] while ^18^EF5, an imidazole derivative, has been used to identify hypoxic regions in plaques in two separate atherosclerotic mouse models [49]. Elevated levels of ^64^Cu-ATSM uptake have similarly been observed in both mouse [57••] and rabbit models [58] of atherosclerosis, demonstrating the potential for hypoxia imaging in this application. Indeed, recent work has reported specific uptake of a nitroimidazole analogue, ^18^F-HX4, as well as ^18^FMISO, in regions of atheromatous plaque in patients with carotid artery stenosis [47, 48••] (Fig. 3). While the accumulating evidence for a role of hypoxia imaging in the early identification of plaque is encouraging, the causality between plaque hypoxia and vulnerability has yet to be conclusively confirmed. A greater understanding of this relationship would significantly aid the development and selection of a suitable hypoxia imaging agent.

## Conclusions and Future Perspectives

Hypoxia can be considered a continuum of injury, both in terms of severity and time, and not an on/off switch. Its definition may therefore shift significantly depending upon our viewpoint or application. As such, we must be specific when we define hypoxia, and equally specific when we screen and validate hypoxia imaging agents for a particular purpose. To optimally exploit our imaging agents, we must fully characterize the degree of hypoxia that they respond to and understand what their retention within a tissue means biologically. With a variety of potential imaging applications characterized by a wide degree of oxygen tensions (and/or oxygen deficits), it is unlikely that there will ever be a “one size fits all” hypoxia imaging agent. In this regard, the flexibility of chemistry in the bis(thiosemicarbazone) compounds represents a significant strength, in that they are highly tunable in terms of hypoxia selectivity and pharmacokinetics, such that they provide the possibility of developing a library of structurally related compounds to be used for a variety of pathologies. Selecting the most appropriate tracer for a given application will require careful co-validation and characterization of each disease process in turn. The next exciting technological development in this field is, perhaps, the increasing availability of PET/MR technology; while MR cannot compete with PET in terms of sensitivity, it may have a significant part to play in terms of providing context for PET hypoxia imaging; enabling parallel real-time indices of tissue perfusion, energetics, and functional response against which hypoxia imaging agents could be validated and corroborated.

## Abbreviations

CVD: Cardiovascular disease
CMVD: Coronary microvascular dysfunction
HIF-1α: Hypoxia inducible factor-1α
PET: Positron emission tomography
SPECT: Single photon emission computed tomography
MRI: Magnetic resonance imaging
BOLD MRI: Blood oxygen level-dependent magnetic resonance imaging
Cu-BTSC: Copper bis(thiosemicarbzone)
^18^FMISO: ^18^F-fluoromisonidazole
LAD: Left anterior descending coronary artery
PCr: Phosphocreatine

## References

[1] De Boer RA, Pinto YM, Van Veldhuisen DJ (2003). The imbalance between oxygen demand and supply as a potential mechanism in the pathophysiology of heart failure: the role of microvascular growth and abnormalities. Microcirculation.

[2] Lanza GA, Crea F (2010). Primary coronary microvascular dysfunction: clinical presentation, pathophysiology, and management. Circulation.

[3] Giordano FJ (2005). Oxygen oxidative stress, hypoxia, and heart failure. J Clin Invest.

[4] Semenza GL (2014). Hypoxia-inducible factor 1 and cardiovascular disease. Annu Rev Physiol.

[5] Hilfiker-Kleiner D, Landmesser U, Drexler H (2006). Molecular mechanisms in heart failure: focus on cardiac hypertrophy, inflammation, angiogenesis, and apoptosis. J Am Coll Cardiol.

[6] Leung DY, Leung M (2011). Non-invasive/invasive imaging: significance and assessment of coronary microvascular dysfunction. Heart.

[7] Camici PG, Crea F (2007). Coronary microvascular dysfunction. N Engl J Med.

[8] Cohn JN, Quyyumi AA, Hollenberg NK, Jamerson KA (2004). Surrogate markers for cardiovascular disease: functional markers. Circulation.

[9] Schachinger V, Britten MB, Zeiher AM (2000). Prognostic impact of coronary vasodilator dysfunction on adverse long-term outcome of coronary heart disease. Circulation.

[10] Owan TE, Hodge DO, Herges RM, Jacobsen SJ, Roger VL, Redfield MM (2006). Trends in prevalence and outcome of heart failure with preserved ejection fraction. N Engl J Med.

[11] Borlaug BA (2014). The pathophysiology of heart failure with preserved ejection fraction. Nat Rev Cardiol.

[12] Oktay AA, Shah SJ (2015). Diagnosis and management of heart failure with preserved ejection fraction: 10 key lessons. Curr Cardiol Rev.

[13] Giamouzis G, Schelbert EB, Butler J (2016). Growing evidence linking microvascular dysfunction with heart failure with preserved ejection fraction. J Am Heart Assoc.

[14] Kato S, Saito N, Kirigaya H, Gyotoku D, Iinuma N, Kusakawa Y, Iguchi K, Nakachi T, Fukui K, Futaki M, Iwasawa T, Kimura K, Umemura S (2016). Impairment of coronary flow reserve evaluated by phase contrast cine-magnetic resonance imaging in patients with heart failure with preserved ejection fraction. J Am Heart Assoc.

[15] Sinusas AJ (1999). The potential for myocardial imaging with hypoxia markers. Semin Nucl Med.

[16] Li D, Dhawale P, Rubin PJ, Haacke EM, Gropler RJ (1996). Myocardial signal response to dipyridamole and dobutamine: demonstration of the BOLD effect using a double-echo gradient-echo sequence. Magn Reson Med.

[17] Huang TY, Liu YJ, Stemmer A, Poncelet BP (2007). T2 measurement of the human myocardium using a T2-prepared transient-state TrueFISP sequence. Magn Reson Med.

[18] Seddon BM, Honess DJ, Vojnovic B, Tozer GM, Workman P. Measurement of tumor oxygenation: in vivo comparison of a luminescence fiber-optic sensor and a polarographic electrode in the p22 tumor. Radiat Res. 2001;155(6):837–46. https://doi.org/10.1667/0033-7587(2001)155[0837:MOTOIV]2.0.CO;2. 10.1667/0033-7587(2001)155[0837:motoiv]2.0.co;2 11352767

[19] Tran LB, Bol A, Labar D, Jordan B, Magat J, Mignion L (2012). Hypoxia imaging with the nitroimidazole 18F-FAZA PET tracer: a comparison with OxyLite, EPR oximetry and 19F-MRI relaxometry. Radiother Oncol.

[20] Machac J (2005). Cardiac positron emission tomography imaging. Semin Nucl Med.

[21] Kudo T (2007). Metabolic imaging using PET. Eur J Nucl Med Mol Imaging.

[22] Coort SL, Bonen A, van der Vusse GJ, Glatz JF, Luiken JJ (2007). Cardiac substrate uptake and metabolism in obesity and type-2 diabetes: role of sarcolemmal substrate transporters. Mol Cell Biochem.

[23] Southworth R (2009). Hexokinase-mitochondrial interaction in cardiac tissue: implications for cardiac glucose uptake, the 18FDG lumped constant and cardiac protection. J Bioenerg Biomembr.

[24] Feher A, Sinusas AJ (2017). Quantitative assessment of coronary microvascular function: dynamic single-photon emission computed tomography, positron emission tomography, ultrasound, computed tomography, and magnetic resonance imaging. Circ Cardiovasc Imaging.

[25] Handley MG, Medina RA, Nagel E, Blower PJ, Southworth R (2011). PET imaging of cardiac hypoxia: opportunities and challenges. J Mol Cell Cardiol.

[26] Valencia A, Burgess JH (1969). Arterial hypoxemia following acute myocardial infarction. Circulation.

[27] Des Tombe AL, Van Beek-Harmsen BJ, Lee-De Groot MB, Van Der Laarse WJ (2002). Calibrated histochemistry applied to oxygen supply and demand in hypertrophied rat myocardium. Microsc Res Tech.

[28] Krohn KA, Link JM, Mason RP (2008). Molecular imaging of hypoxia. J Nucl Med.

[29] Strauss HW, Nunn A, Linder K (1995). Nitroimidazoles for imaging hypoxic myocardium. J Nucl Cardiol.

[30] Nunn A, Linder K, Strauss HW (1995). Nitroimidazoles and imaging hypoxia. Eur J Nucl Med.

[31] Dearling JL, Lewis JS, Mullen GE, Welch MJ, Blower PJ (2002). Copper bis(thiosemicarbazone) complexes as hypoxia imaging agents: structure-activity relationships. J Biol Inorg Chem: JBIC.

[32] Fujibayashi Y, Taniuchi H, Yonekura Y, Ohtani H, Konishi J, Yokoyama A (1997). Copper-62-ATSM: a new hypoxia imaging agent with high membrane permeability and low redox potential. J Nucl Med.

[33] Chia K, Fleming IN, Blower PJ (2012). Hypoxia imaging with PET: which tracers and why?. Nucl Med Commun.

[34] Sutherland FJ, Hearse DJ (2000). The isolated blood and perfusion fluid perfused heart. Pharmacol Res.

[35] Yabe T, Mitsunami K, Inubushi T, Kinoshita M (1995). Quantitative measurements of cardiac phosphorus metabolites in coronary artery disease by 31P magnetic resonance spectroscopy. Circulation.

[36] Buchthal SD, den Hollander JA, Merz CN, Rogers WJ, Pepine CJ, Reichek N (2000). Abnormal myocardial phosphorus-31 nuclear magnetic resonance spectroscopy in women with chest pain but normal coronary angiograms. N Engl J Med.

[37] Smith CS, Bottomley PA, Schulman SP, Gerstenblith G, Weiss RG (2006). Altered creatine kinase adenosine triphosphate kinetics in failing hypertrophied human myocardium. Circulation.

[38] Rankin EB, Giaccia AJ (2016). Hypoxic control of metastasis. Science.

[39] Barker HE, Paget JT, Khan AA, Harrington KJ (2015). The tumour microenvironment after radiotherapy: mechanisms of resistance and recurrence. Nat Rev Cancer.

[40] Ballinger JR (2001). Imaging hypoxia in tumors. Semin Nucl Med.

[41] Rasey JS, Koh WJ, Evans ML, Peterson LM, Lewellen TK, Graham MM (1996). Quantifying regional hypoxia in human tumors with positron emission tomography of [18F]fluoromisonidazole: a pretherapy study of 37 patients. Int J Radiat Oncol Biol Phys.

[42] Martin GV, Cerqueira MD, Caldwell JH, Rasey JS, Embree L, Krohn KA (1990). Fluoromisonidazole. A metabolic marker of myocyte hypoxia. Circ Res.

[43] Shelton ME, Dence CS, Hwang DR, Welch MJ, Bergmann SR (1989). Myocardial kinetics of fluorine-18 misonidazole: a marker of hypoxic myocardium. J Nucl Med.

[44] Shelton ME, Dence CS, Hwang DR, Herrero P, Welch MJ, Bergmann SR (1990). In vivo delineation of myocardial hypoxia during coronary occlusion using fluorine-18 fluoromisonidazole and positron emission tomography: a potential approach for identification of jeopardized myocardium. J Am Coll Cardiol.

[45] Mateo J, Izquierdo-Garcia D, Badimon JJ, Fayad ZA, Fuster V (2014). Noninvasive assessment of hypoxia in rabbit advanced atherosclerosis using (1)(8)F-fluoromisonidazole positron emission tomographic imaging. Circ Cardiovasc Imaging.

[46] Manabe O, Hirata K, Shozo O, Shiga T, Uchiyama Y, Kobayashi K, Watanabe S, Toyonaga T, Kikuchi H, Oyama-Manabe N, Tamaki N (2017). 18F-fluoromisonidazole (FMISO) PET may have the potential to detect cardiac sarcoidosis. J Nucl Cardiol.

[47] Joshi FR, Manavaki R, Fryer TD, Figg NL, Sluimer JC, Aigbirhio FI, Davenport AP, Kirkpatrick PJ, Warburton EA, Rudd JHF (2017). Vascular imaging with 18F-fluorodeoxyglucose positron emission tomography is influenced by hypoxia. J Am Coll Cardiol.

[48] van der Valk FM, Sluimer JC, Voo SA, Verberne HJ, Nederveen AJ, Windhorst AD (2015). In vivo imaging of hypoxia in atherosclerotic plaques in humans. JACC Cardiovasc Imaging.

[49] Silvola JM, Saraste A, Forsback S, Laine VJ, Saukko P, Heinonen SE (2011). Detection of hypoxia by [18F]EF5 in atherosclerotic plaques in mice. Arterioscler Thromb Vasc Biol.

[50] Medina RA, Mariotti E, Pavlovic D, Shaw KP, Eykyn TR, Blower PJ (2015). 64Cu-CTS: a promising radiopharmaceutical for the identification of low-grade cardiac hypoxia by PET. J Nucl Med.

[51] Handley MG, Medina RA, Mariotti E, Kenny GD, Shaw KP, Yan R (2014). Cardiac hypoxia imaging: second-generation analogues of 64Cu-ATSM. J Nucl Med.

[52] Handley MG, Medina RA, Paul RL, Blower PJ, Southworth R (2013). Demonstration of the retention of 64Cu-ATSM in cardiac myocytes using a novel incubation chamber for screening hypoxia-dependent radiotracers. Nucl Med Commun.

[53] Fujibayashi Y, Cutler CS, Anderson CJ, McCarthy DW, Jones LA, Sharp T, Yonekura Y, Welch MJ (1999). Comparative studies of Cu-64-ATSM and C-11-acetate in an acute myocardial infarction model: ex vivo imaging of hypoxia in rats. Nucl Med Biol.

[54] Shaughnessy F, Mariotti E, Shaw KP, Eykyn TR, Blower PJ, Siow R (2014). Modification of intracellular glutathione status does not change the cardiac trapping of (64)Cu(ATSM). EJNMMI Res.

[55] Wada K, Fujibayashi Y, Tajima N, Yokoyama A (1994). Cu-ATSM, an intracellular-accessible superoxide dismutase (SOD)-like copper complex: evaluation in an ischemia-reperfusion injury model. Biol Pharm Bull.

[56] Takahashi N, Fujibayashi Y, Yonekura Y, Welch MJ, Waki A, Tsuchida T, Sadato N, Sugimoto K, Nakano A, Lee JD, Itoh H (2001). Copper-62 ATSM as a hypoxic tissue tracer in myocardial ischemia. Ann Nucl Med.

[57] Nie X, Randolph GJ, Elvington A, Bandara N, Zheleznyak A, Gropler RJ (2016). Imaging of hypoxia in mouse atherosclerotic plaques with (64)Cu-ATSM. Nucl Med Biol.

[58] Nie X, Laforest R, Elvington A, Randolph GJ, Zheng J, Voller T, Abendschein DR, Lapi SE, Woodard PK (2016). PET/MRI of hypoxic atherosclerosis using 64Cu-ATSM in a rabbit model. J Nucl Med.

[59] Lee ST, Scott AM (2007). Hypoxia positron emission tomography imaging with 18f-fluoromisonidazole. Semin Nucl Med.

[60] Peeters SG, Zegers CM, Lieuwes NG, van Elmpt W, Eriksson J, van Dongen GA (2015). A comparative study of the hypoxia PET tracers [(1)(8)F]HX4, [(1)(8)F]FAZA, and [(1)(8)F]FMISO in a preclinical tumor model. Int J Radiat Oncol Biol Phys.

[61] Fujibayashi Y, Matsumoto K, Yonekura Y, Konishi J, Yokoyama A (1989). A new zinc-62/copper-62 generator as a copper-62 source for PET radiopharmaceuticals. J Nucl Med.

[62] Dearling JL, Packard AB (2010). Some thoughts on the mechanism of cellular trapping of Cu(II)-ATSM. Nucl Med Biol.

[63] Maurer RI, Blower PJ, Dilworth JR, Reynolds CA, Zheng Y, Mullen GE (2002). Studies on the mechanism of hypoxic selectivity in copper bis(thiosemicarbazone) radiopharmaceuticals. J Med Chem.

[64] Paterson BM, Donnelly PS (2011). Copper complexes of bis(thiosemicarbazones): from chemotherapeutics to diagnostic and therapeutic radiopharmaceuticals. Chem Soc Rev.

[65] Yip C, Blower PJ, Goh V, Landau DB, Cook GJ (2015). Molecular imaging of hypoxia in non-small-cell lung cancer. Eur J Nucl Med Mol Imaging.

[66] McQuade P, Martin KE, Castle TC, Went MJ, Blower PJ, Welch MJ, Lewis JS (2005). Investigation into 64Cu-labeled Bis(selenosemicarbazone) and Bis(thiosemicarbazone) complexes as hypoxia imaging agents. Nucl Med Biol.

[67] Dearling JL, Lewis JS, Mullen GE, Rae MT, Zweit J, Blower PJ (1998). Design of hypoxia-targeting radiopharmaceuticals: selective uptake of copper-64 complexes in hypoxic cells in vitro. Eur J Nucl Med.

[68] Brown OC, Baguna Torres J, Holt KB, Blower PJ, Went MJ (2017). Copper complexes with dissymmetrically substituted bis(thiosemicarbazone) ligands as a basis for PET radiopharmaceuticals: control of redox potential and lipophilicity. Dalton Trans.

[69] Tarkin JM, Joshi FR, Rudd JH (2014). PET imaging of inflammation in atherosclerosis. Nat Rev Cardiol.

[70] Moss AJ, Adamson PD, Newby DE, Dweck MR (2016). Positron emission tomography imaging of coronary atherosclerosis. Futur Cardiol.

[71] Joseph P, Tawakol A (2016). Imaging atherosclerosis with positron emission tomography. Eur Heart J.

[72] Ross R (1999). Atherosclerosis—an inflammatory disease. N Engl J Med.

[73] Libby P, Ridker PM, Maseri A (2002). Inflammation and atherosclerosis. Circulation.

[74] Rudd JH, Warburton EA, Fryer TD, Jones HA, Clark JC, Antoun N, Johnström P, Davenport AP, Kirkpatrick PJ, Arch BN, Pickard JD, Weissberg PL (2002). Imaging atherosclerotic plaque inflammation with [18F]-fluorodeoxyglucose positron emission tomography. Circulation.

[75] Tawakol A, Migrino RQ, Bashian GG, Bedri S, Vermylen D, Cury RC, Yates D, LaMuraglia GM, Furie K, Houser S, Gewirtz H, Muller JE, Brady TJ, Fischman AJ (2006). In vivo 18F-fluorodeoxyglucose positron emission tomography imaging provides a noninvasive measure of carotid plaque inflammation in patients. J Am Coll Cardiol.

[76] Rudd JH, Narula J, Strauss HW, Virmani R, Machac J, Klimas M (2010). Imaging atherosclerotic plaque inflammation by fluorodeoxyglucose with positron emission tomography: ready for prime time?. J Am Coll Cardiol.

[77] Williams G, Kolodny GM (2008). Suppression of myocardial 18F-FDG uptake by preparing patients with a high-fat, low-carbohydrate diet. AJR Am J Roentgenol.

[78] de Groot M, Meeuwis AP, Kok PJ, Corstens FH, Oyen WJ (2005). Influence of blood glucose level, age and fasting period on non-pathological FDG uptake in heart and gut. Eur J Nucl Med Mol Imaging.

[79] Rogers IS, Nasir K, Figueroa AL, Cury RC, Hoffmann U, Vermylen DA, Brady TJ, Tawakol A (2010). Feasibility of FDG imaging of the coronary arteries: comparison between acute coronary syndrome and stable angina. JACC Cardiovasc Imaging.

[80] Heughan C, Niinikoski J, Hunt TK (1973). Oxygen tensions in lesions of experimental atherosclerosis of rabbits. Atherosclerosis.

[81] Bjornheden T, Levin M, Evaldsson M, Wiklund O (1999). Evidence of hypoxic areas within the arterial wall in vivo. Arterioscler Thromb Vasc Biol.

[82] Sluimer JC, Gasc JM, van Wanroij JL, Kisters N, Groeneweg M, Sollewijn Gelpke MD, Cleutjens JP, van den Akker LH, Corvol P, Wouters BG, Daemen MJ, Bijnens APJ (2008). Hypoxia, hypoxia-inducible transcription factor, and macrophages in human atherosclerotic plaques are correlated with intraplaque angiogenesis. J Am Coll Cardiol.

[83] Folco EJ, Sheikine Y, Rocha VZ, Christen T, Shvartz E, Sukhova GK (2011). Hypoxia but not inflammation augments glucose uptake in human macrophages: implications for imaging atherosclerosis with 18fluorine-labeled 2-deoxy-D-glucose positron emission tomography. J Am Coll Cardiol.

